# Non-destructive monitoring of amylose content in rice by UAV-based hyperspectral images

**DOI:** 10.3389/fpls.2022.1035379

**Published:** 2022-10-27

**Authors:** Fumin Wang, Qiuxiang Yi, Lili Xie, Xiaoping Yao, Jueyi Zheng, Tianyue Xu, Jiale Li, Siting Chen

**Affiliations:** ^1^ Institute of Applied Remote Sensing & Information Technology, Zhejiang University, Hangzhou, China; ^2^ Key Laboratory of Agricultural Remote Sensing and Information System, Zhejiang University, Hangzhou, China; ^3^ State Key Laboratory of Desert and Oasis Ecology, Xinjiang Institute of Ecology and Geography, Chinese Academy of Sciences, Urumqi, China

**Keywords:** amylose content, rice, UAV-based hyperspectral images, spectral variables, textural measures

## Abstract

Amylose content (AC) is an important indicator for rice quality grading. The rapid development of unmanned aerial vehicle (UAV) technology provides rich spectral and spatial information on observed objects, making non-destructive monitoring of crop quality possible. To test the potential of UAV-based hyperspectral images in AC estimation, in this study, observations on five rice cultivars were carried out in eastern China (Zhejiang province) for four consecutive years (from 2017 to 2020). The correlations between spectral and textural variables of UAV-based hyperspectral images at different growth stages (booting, heading, filling, and ripening) and AC (%) were analyzed, and the linear regression models based on spectral variables alone, textural variables alone, and combined spectral and textural variables were established. The results showed that the sensitive bands (P< 0.001) to AC were mainly centered in the green (536∽568 nm) and red regions (630∽660nm), with spectral and textural variables at the ripening stage giving the highest negative correlation coefficient of -0.868 and -0.824, respectively. Models based on combined spectral and textural variables give better estimation than those based on spectral or textural variables alone, characterized by less variables and higher accuracy. The best models using spectral or textural variables alone both involved three growth stages (heading, filling, and ripening), with root mean square error (RMSE) of 1.01% and 1.04%, respectively, while the models based on combined spectral and textural variables have RMSE of 1.04% 0.844% with only one (ripening stage) or two (ripening and filling stages) growth stages involved. The combination of spectral and textural variables of UAV-based hyperspectral images is expected to simplify data acquisition and enhance estimation accuracy in remote sensing of rice AC.

## Introduction

As the main staple food for over half of the world’s population, rice is one of the most important food crops in the world and its quality is especially important (Yang and Wang, 2019). Amylose content (AC), combined with grain shape and gelatinization temperature, are three criteria for rice market classes in the United States and can also be used for rice cultivar categorization (Bergman, 2019). Furthermore, AC is the most important factor defining the palatability or specialty type of rice (Li et al., 2016), and thus the faster measurement of AC draws a lot of attention from scientists and technicians who are interested in food quality.

Due to the time-consuming, destructive, laborious, and complicated analysis procedure (Caporaso et al., 2021), chemical methods for AC measurement in the laboratory, such as the iodine colorimetric method (Williams et al., 1958) and size-exclusion chromatography (Fitzgerald et al., 2009), are unable to meet the requirement of rapid determination of rice AC for quality grading or variety classification. Methods featured with non-contact and rapid estimation using biomaterial optical properties obtained by spectroscopy or imaging systems are widely used for the measurement of crop quality traits (ElMasry et al., 2019).

Most studies on non-destructive estimation of AC by spectral information have adopted such analytical techniques as near-infrared (NIR) spectroscopy (Bagchi et al., 2016; Díaz et al., 2019) and hyperspectral imaging (HSI) (Caporaso et al., 2018; Huang et al., 2021) to collect spectral information of samples in the shape of milled rice flour (Bao et al., 2001; Wu and Shi, 2004), brown rice flour (Shu et al., 1999), milled whole grain (Windham et al., 1997), brown rice (Bagchi et al., 2016; Díaz et al., 2019), etc., which were scanned in reflectance or transmittance mode on a moving platform. In NIR or HSI methods, samples used for developing and validating calibration equations are generally collected from harvested rough rice, in this case, the differences in amylose content caused by environmental factors are not considered in spectral information. However, the AC of each sample is a consequence of a unique combination of genetic and environmental effects (Bergman, 2019), and the formation of amylose is strongly affected by ambient air temperature (Díaz et al., 2019). Juliano and Pascual (1980) demonstrated that the same cultivar grown in different environments may vary by up to 6% in AC. Some research also showed that low-amylose types, which typically have 12%–15% AC when grown at higher temperatures, have up to 18% when grown at lower temperatures (Larkin and Park, 1999; Bao et al., 2000). This means that additional analysis is required to update the calibration equation to function with each new set of samples, even for samples coming from the same cultivar. Such analysis is generally expensive and time-consuming.

Is it possible to obtain knowledge about rice AC from spectral and spatial information of stand crops in the field before harvest? This can not only help rice breeders to obtain estimates of a cultivar’s AC rapidly to assist in classifying rice cultivars but also provide opportunities to manage rice harvest differently (Basnet et al., 2003) and further get helpful information for rice market classing in advance. In fact, many researchers have used remote sensed data over large regions to predict parameters that relate to crop quality, such as grain protein content (Ryu et al., 2011; Chen, 2020; Li et al., 2020). By far, there is no relevant research of any attempt to relate rice AC with remote-sensing spectral or spatial information in hyperspectral images of rice plants.

With the characteristics of high spatial resolution, high temporal resolution, and easy operation, unmanned aerial vehicles (UAVs) have been used as a new technical means for monitoring physiological and biochemical parameters of crops in fields rapidly and non-destructively. Progress has been made in using UAV-based multispectral or hyperspectral images to estimate agricultural parameters such as grain yield (Wang et al., 2021), leaf area index (LAI) (Roosjen et al., 2018), chlorophyll (Tewes and Schellberg, 2018), and nitrogen (N) (Zheng et al., 2020). In addition to various spectral information, UAV-based images generally have spatial resolution at the centimeter level and thus can provide rich spatial information about observed objects. Some research has proven the great potential of textural information in UAV-based images for crop parameter monitoring, such as wheat biomass (Yue et al., 2019), rice N content (Zheng et al., 2020), and grain yield (Wang et al., 2021). In recent years, with increased accessibility, reduced sensor costs, and the speedy development in technology for data processing, UAVs have become a widely used means for agronomic trait monitoring (Deng et al., 2018).

In view of the abovementioned background, the main objectives of this study are to 1) identify the bands of UAV hyperspectral images that are sensitive to rice AC and the crucial growth stages for AC estimation and 2) test the feasibility of using spectral and textural features in UAV hyperspectral images for rice AC estimation.

## Materials and methods

### Study area

**Figure 1:**
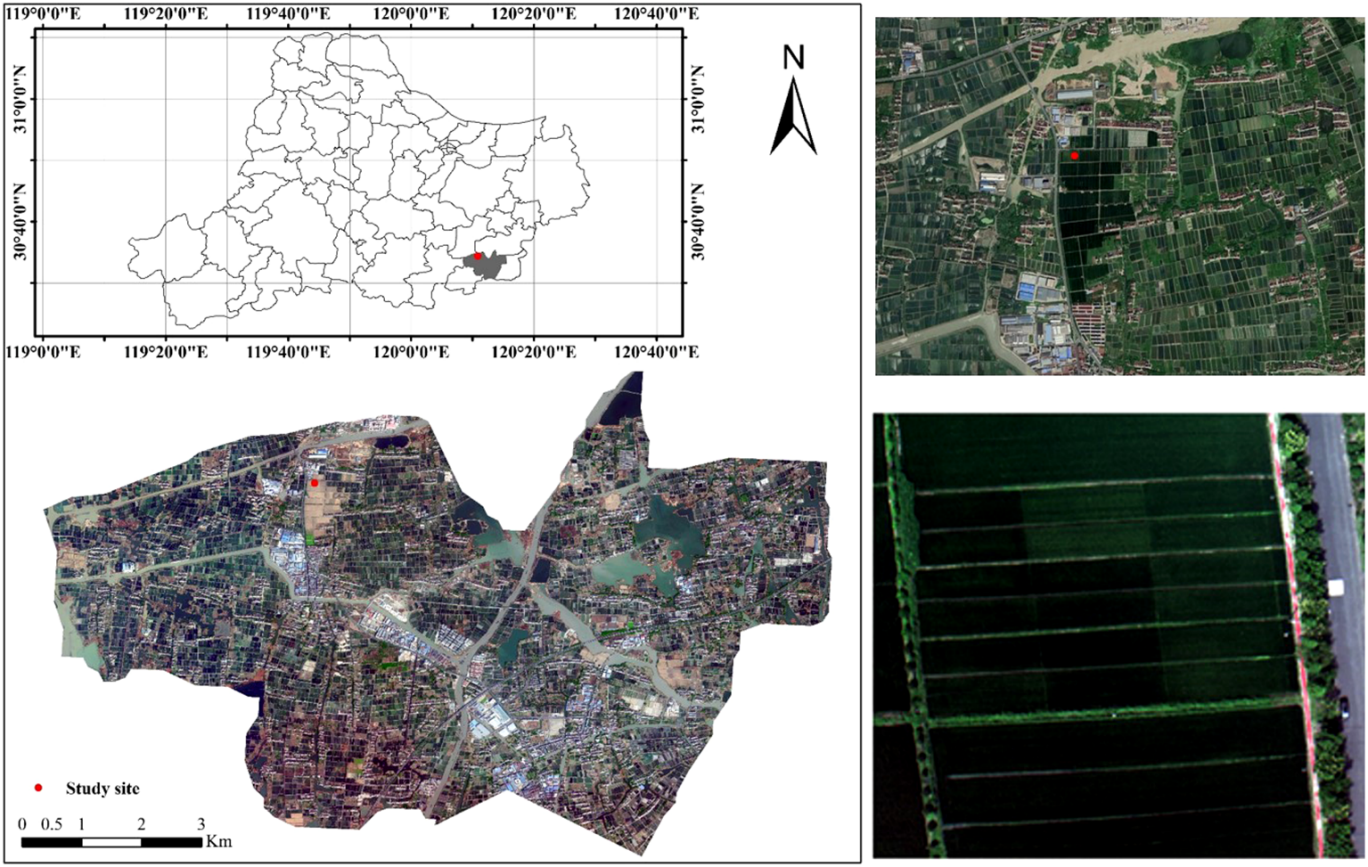
Study area and experimental site.

The study area (30°26∼30°42, 119°45’∼120°21’) is located at Xiashe village, Deqing County, Zhejiang Province (**Figure 1**). The annual climate here is characterized by four distinct seasons, sufficient sunlight, abundant rainfall, with the annual average temperature ranging from 13°C to 16°C, and the annual average precipitation exceeding 1,300 mm. Xiashe village, as the first batch of provincial-level functional areas for grain production in Deqing County, has advanced planting technology and scientific management for rice, making the accuracy of experimental data stable.

### Field experiment

The experimental site covers an area of about 0.82 ha (81 m * 101 m) and is divided into 20 plots according to different combinations of rice varieties and N fertilization levels. Field campaigns for data acquisition were carried out for four consecutive years (from 2017 to 2020) with two rice cultivars and five N fertilization levels set for each year. The selected varieties for each experimental year are in line with the most widely planted varieties in the local area, consisting of Zhegeng 99 and Jia 58 in 2017, Nangeng 9108 and Nangeng 46 in 2019, and Zhegeng 99 and Jia 67 in 2018 and 2020. Rice seeds were generally sown in mid to late May, transplanted in early to middle June, and harvested in late November, with the whole growth length about 153–165 days. Five nitrogen (N) rates (N0-N4: 0, 112.5-142.5, 225-285, 337.5-427.5, 450-570 kg ha-1) with the same amount of phosphate (75 kg ha-1) and potash (150 kg ha-1) fertilizer were set. All treatments were composed of two or more repeated plots.

### Data acquisition

#### Determination of amylose content

During the maturing stage, three 75 cm × 75 cm quadrats were randomly selected from each plot to obtain rice samples. After sampling, the samples were first dried in an oven for 40 min at 105°C and then dried to a constant weight at 65°C. Samples of rice, rice grains, and rice flour were obtained through threshing, shelling, and milling procedures. AC (%) in milled rice flour samples of each plot was determined in the laboratory at China National Rice Research Institute using a spectrophotometry method (NY/T2639-2014).

#### UAV-based hyperspectral image acquisition

At the key growth stages for rice quality formation, the campaigns for UAV hyperspectral image acquisition were carried out on clear and cloudless days between 10:00 a.m. and 2:00 p.m. local time. The specific dates for image acquisition from 2017 to 2020 are listed in **Table 1**.

**Table 1:** Dates for unmanned aerial vehicle (UAV)-based hyperspectral image acquisition.

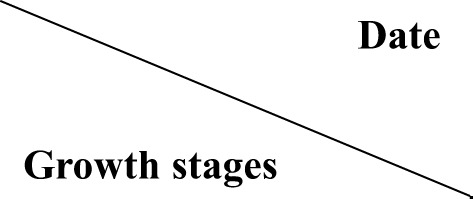	2017	2018	2019		2020	
			Variety1	Variety2	Variety1	Variety2
**Booting**	27/08	23/08	29/07	11/08	24/08	27/08
**Heading**	08/09	08/09	11/08	20/08	01/09	05/09
**Filling**	19/09	24/09	20/08	30/08	12/09	24/09
**Ripening**	03/11	09/11	30/09	10/10	20/10	30/10

Hyperspectral images were collected by a six-rotor UAV (DJI M600 Pro) mounted with a hyperspectral imager (Rikola). Featured with the high load and excellent flight performance, DJI M600 Pro six-rotor UAV adopts a modular design to further improve reliability and convenience. It has a maximum takeoff weight of 15.5 kg, a maximum payload of 6 kg, and a maximum flight range of 5 km. During flights, the UAV platform was also equipped with the Ronin-MX stabilization gimbal to maintain the stability of the hyperspectral imager and reduce the disturbance of aircraft shaking and wind disturbance, so as to ensure the high quality of obtained hyperspectral images. The flight altitude was fixed at 200 m, with a ground spatial resolution of 0.13 m. Three fixed points were set on the flight line; each had a hovering shooting time of 45 s.

The hyperspectral imager Rikola carried by the UAV platform is a frame-type hyperspectral imager that not only can be used for handheld measurement but also is suitable for the UAV platform. The default band range of this imager is 500–900 nm, and the number of bands was set at 62 in this study. The central band and full width at half maximum (FWHM) of these bands are shown in **Table 2**.

**Table 2:** Central wavelength (WL) and full width at half maximum (FWHM) of the used hyperspectral imager.

WL (nm)	FWHM (nm)	WL (nm)	FWHM (nm)	WL (nm)	FWHM (nm)	WL (nm)	FWHM (nm)	WL (nm)	FWHM (nm)
512	8.18	596	7.72	685	5.82	736	6.68	824	7.69
520	8.51	600	7.76	688	6.06	740	6.14	832	13.62
528	7.58	604	7.26	692	6.50	744	6.23	840	14.49
536	8.55	608	9.24	696	7.45	748	5.87	848	13.53
544	7.63	616	10.21	700	6.99	752	6.59	856	13.61
552	7.77	624	9.20	704	5.89	760	5.96	864	12.42
560	7.98	632	8.93	709	6.00	768	6.09	872	12.75
568	7.27	635	9.29	712	6.44	776	8.71	880	12.83
576	8.30	650	8.36	716	6.91	784	7.38	888	10.51
580	8.16	656	9.37	720	6.86	792	8.51		
584	7.24	664	8.75	724	6.22	800	8.72		
588	6.51	672	9.43	728	5.81	808	8.16		
592	7.24	680	9.80	733	6.39	816	9.10		

### Image processing

Data format conversion, lens correction, geometric halo correction, and dark current correction of acquired hyperspectral images were realized through the corresponding module function of Hyperspectral Imager V2.1.4 (Rikola, Ltd.) software, which is built-in with the camera. Through these operations, hyperspectral image data can be transformed into editable, clear, and accurate radiation images. Band registration was performed by RegMosaic (Rikola, Ltd.) software. The radiometric correction was conducted by ENVI using the radiation and reflectance of a standard diffuse reflection plate, of which the radiation and reflectance are fixed values. For details about the calculation of image reflectance, refer to Wang et al. (2021).

#### Extraction of textural measures

Gray-level co-occurrence matrix (GLCM), as the most commonly implemented method for textural analysis, was used to extract textural features from UAV-based hyperspectral images in the present study. GLCM is a square matrix with the number of rows and columns that is the same as the gray values in the image. The matrix element contains the second-order statistical probability values for changes between two gray levels at a particular displacement distance and at a particular angle. The displacement distance and moving direction are important to the construction of a GLCM. Here, GLCMs were constructed with the displacement distance of 1 pixel and the moving directions of 0°, 45°, 90°, and 135°, and by doing so eliminates the effect of moving direction on the results. The average value of these four directions was finally used. As each hyperspectral image has 62 bands, 62 GLCMs are generated for each hyperspectral image. In order to quantitatively describe the textural features contained in GLCMs, statistical measures can further be calculated using these conditional probabilities in GLCMs to generate the textural properties. Haralick et al. (1973) proposed a set of textural descriptors based on GLCMs. Eight of the most commonly used textural descriptors were selected in the present work, including angular second moment (ASM), entropy (ENT; the opposite of ASM, high when the pixel values of the GLCM have varying values), homogeneity (HOM), contrast (CON), dissimilarity (DIS), correlation (COR), mean (MEA), and variance (VAR). Through trial and error, a moving square window with a size of 3 pixels × 3 pixels was applied for calculation of textural measures. The formulas and meaning of those eight textural measures also can be found in Park and Guldmann (2020) and Wang et al. (2021). For more information about GLCM, refer to Hall-Beyer (2017) and Park and Guldmann (2020).

#### Construction of spectral and textural indices

The two most widely used types of vegetation index, i.e., ratio vegetation index (RVI) and difference vegetation index (DVI), were adopted for the construction of spectral and textural indices. Spectral indices were calculated using reflectance by two-band combinations of 62 bands in the form of these two types, as were textural indices, except they were based on eight textural measures. The formulas of these two types of vegetation indices were presented in **Table 3**. ENVI 5.3 (Exelis Visual Information Solutions, Inc.) was used for their calculations.

**Table 3:** Formulas of textural and spectral index calculation.

Textural index	Formula	Vegetation index	Formula
RTI [Λ1, Λ2]	TS_Λ1_/TS_Λ2_	RVI [Λ1, Λ2]	R_Λ1_/R_Λ2_
DTI [Λ1, Λ2]	TS_Λ1_-TS_Λ2_	DVI [Λ1, Λ2]	R_Λ1_-R_Λ2_

TS, textural statistics; R, reflectance; Λ1, wavelengths in near-infrared (NIR) region (760–900 nm); Λ2, wavelengths in the RED region (620–760 nm). All two-band combinations between NIR and RED were calculated.

### Model development and evaluation

To verify the capabilities of spectral and textural variables of UAV-based hyperspectral images for the estimation of rice AC, three types of models based on spectral variables alone, textural measures alone, and combined spectral and textural information were developed using the stepwise multiple linear regression method. The expression of the model is as in Eq. 1:


(1) $$AC=a_{1}X_{1}+a_{2}X_{2}+a_{3}X_{3}+\cdots +a_{n}X_{n}+b$$

where AC is the estimated amylose content, *a* is the coefficient of independent variables or the slope, b is the intercept, and *n* is the number of independent variables.

Moreover, three criteria, namely the coefficient of determination (*R^2^*), root mean square error (RMSE), and mean absolute prediction error (MAPE), were used to test the performance of the models.

## Results and discussion

### Statistics of measured amylose content

Two-thirds of 80 measured AC, with the number 54, were randomly selected as calibration data for model development, and the remaining 26 data were used for model validation. The maximum AC was 18.3% and the minimum was 10.0%, with an average AC of 14.14% (**Table 4**). The distribution range of the validation dataset was within that of the calibration dataset.

**Table 4:** Statistics of measured amylose content for the calibration and validation datasets.

Datasets	Number of samples	Range	Mean	SD
***All***	80	10.0~18.3	14.14	2.86
***Calibration set***	54	10.0~18.3	14.14	2.87
***Validation set***	26	10.2~18.0	14.14	2.83

### Estimation of amylose content based on spectral variables alone

#### Relationship between amylose content and spectral reflectance at different growth stages

**Figure 2:**
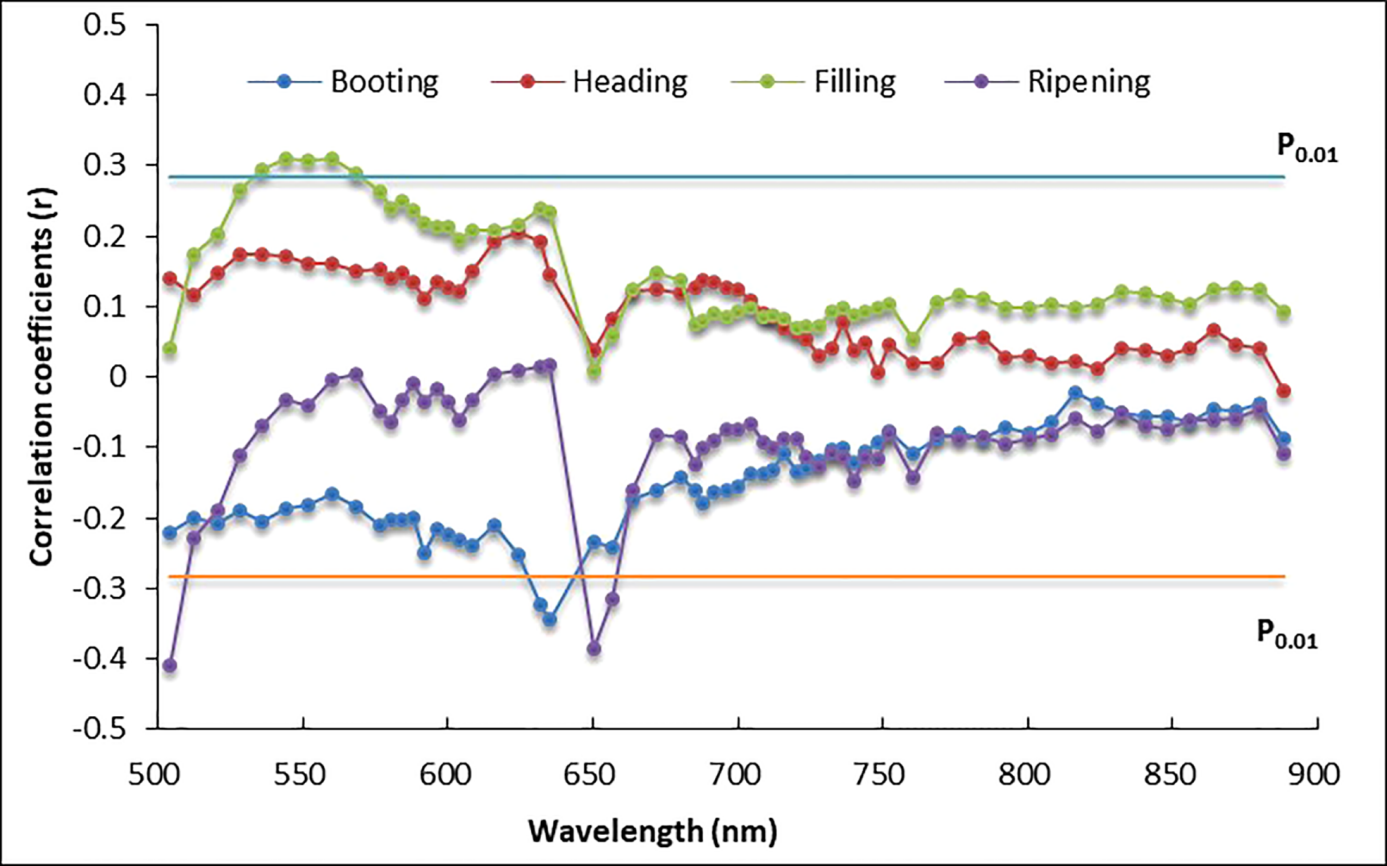
Correlation coefficients between rice amylose content (AC) and reflectance of unmanned aerial vehicle (UAV)-based hyperspectral images.

The relationship between AC and reflectance of UAV hyperspectral images at different growth stages (**Figure 2**) showed that most of the correlation coefficients were below the 1% significance level (when the number of samples *n* = 80, *P*_0.01_ = 0.283), especially those at the heading stage, with all coefficients below the 1% significance level line, showing the lowest correlation with AC compared to the other three growth stages. The reflectance at 504 and 650 nm at the ripening stage had the most significant correlation with AC (*r* = -0.411 and *r* = -0.386), followed by the spectrum at 635 nm at the booting stage (*r* = -0.346), and both showed a negative correlation with AC.

The significant positive correlation was also found for five other spectrum bands (from 536 to 568 nm) at the filling stage, with correlation coefficients (*r*) of approximately 0.3, slightly higher than the 1% significance level. The sensitive bands to AC were mainly centered in the green (536∽568 nm) and red regions (630∽660 nm). Spectrum reflectance in the NIR region at all four growth stages showed poor correlation with AC.

#### Relationship between amylose content and spectral indices at different growth stages

**Figure 3:**
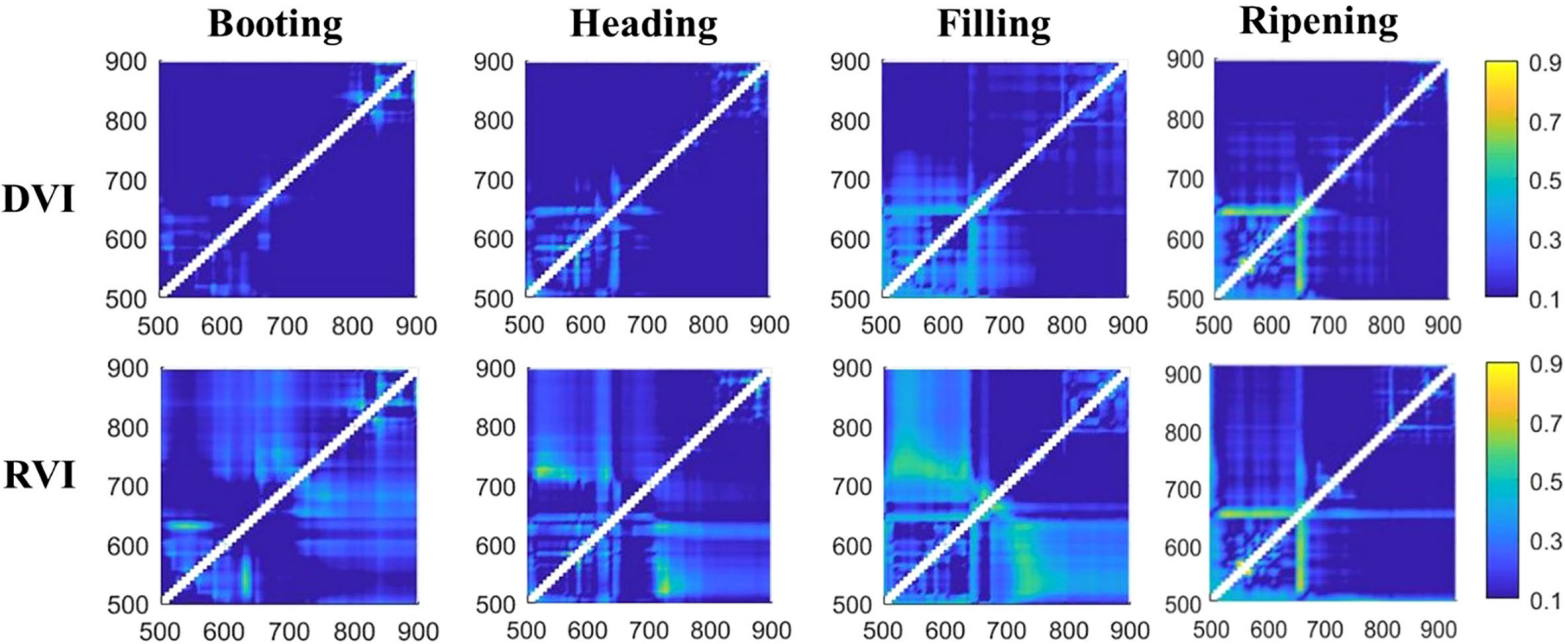
Matrix plots of correlation coefficients for the relationship between difference vegetation index (DVI) vs. amylose content (AC) and ratio vegetation index (RVI) vs. AC at different growth stages.

The matrix plots (**Figure 3**) of correlation coefficients (*r*) for the correlation between AC and two types of vegetation indices, i.e., DVI and RVI, at four growth stages showed that the majority of *r* values were smaller than 0.3, with the major color of the map in blue, indicating the insensitive correlation with AC. However, significant correlation can still be found in some combinations. Further analysis showed that the most significant correlations have *r* values of 0.692~0.795 for DVI and 0.781~0.868 for RVI. The highest *r* of 0.868 was given by the vegetation index in RVI type, which was constructed by the combination of the reflectance in 568 and 580 nm at the ripening stage.

Vegetation indices were significantly better correlated to AC than raw reflectance (**Table 5**), with all correlation coefficients significant at 0.001 probability level (*P*< 0.001), instead of the 0.01 significance level of raw reflectance. Furthermore, vegetation indices in the RVI type showed a higher correlation with AC than the DVI type, and RVI at the ripening stage had the most significant negative correlation with AC, with an *r* value of -0.868. Additionally, the highest correlation coefficients were all given by vegetation indices that were constructed by reflectance at the ripening stage, and only at this stage was the raw reflectance correlated to AC at the 0.001 significance level compared to those at the other three growth stages, demonstrating the great role of the ripening stage for the estimation of AC.

**Table 5:** Correlation coefficients (*r*) of the most sensitive reflectance and vegetation indices to amylose content.

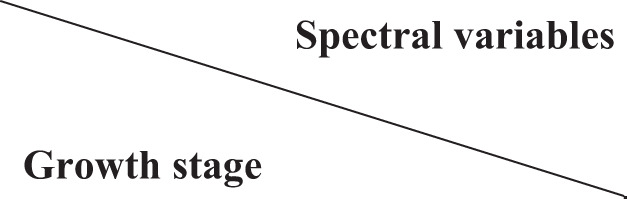	Reflectance	DVI	RVI
Booting stage	-0.346*	0.742**	0.781**
Heading stage	0.206	0.692**	0.842**
Filling stage	0.310*	-0.762**	-0.759**
Ripening stage	-0.386**	0.795**	-0.868**

*denotes significance at the 0.01 probability level (when the number of samples n = 80, P_0.01_ = 0.283), and ** denotes significance at the 0.001 probability level (when the number of samples n = 80, P_0.001_ = 0.356). DVI, difference vegetation index; RVI, ratio vegetation index.

#### Estimation models based on spectral variables alone

**Figure 4:**
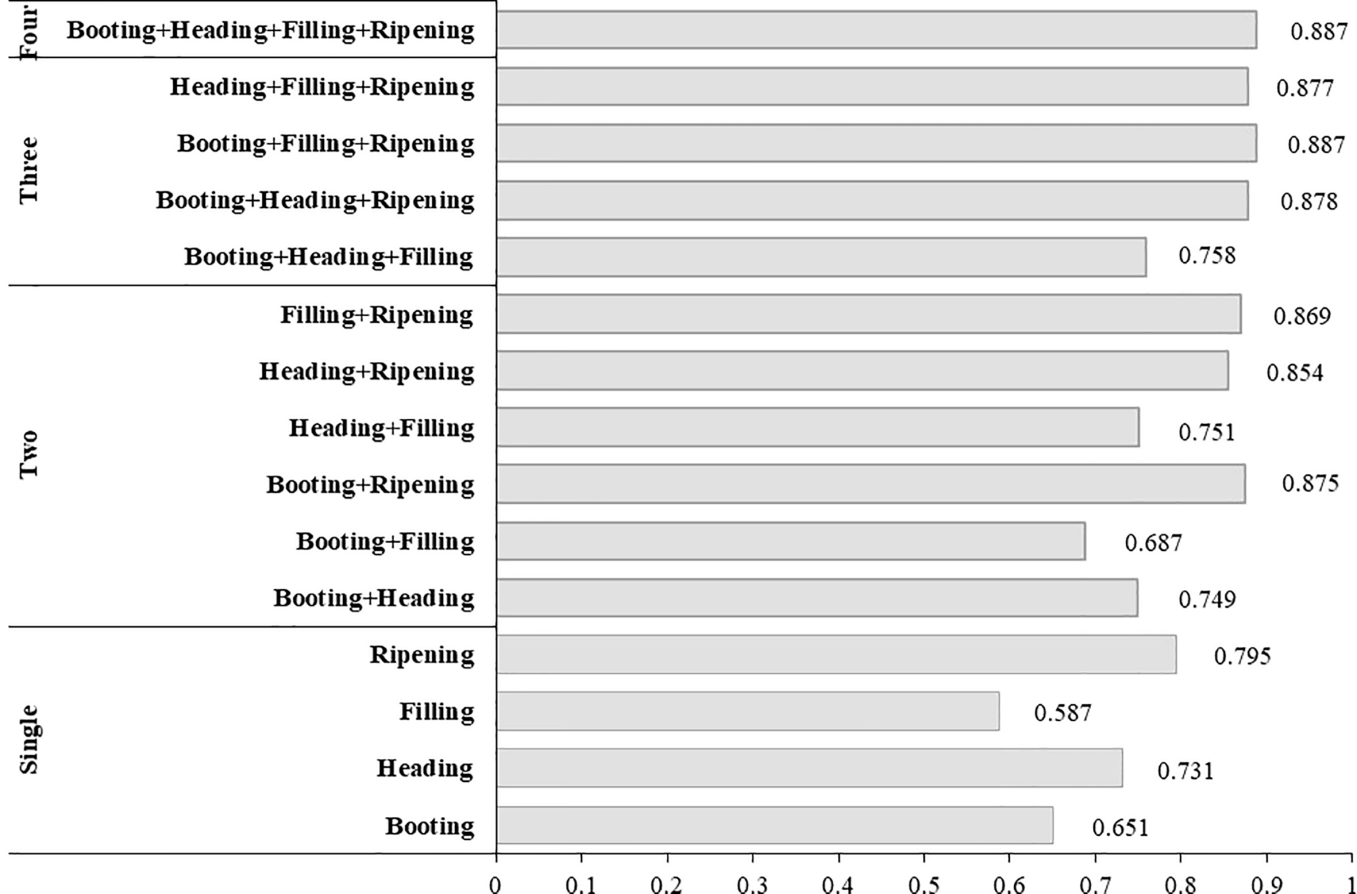
Coefficient of determination (*R^2^*) of regression models for amylose content (AC) estimation based on spectral variables alone.

In order to identify the models that have less variables but higher accuracy for AC estimation, models based on spectral variables at one, two, three, and four growth stages were established, and the coefficients of determination (*R^2^*) for these models were compared (**Figure 4**).

The accuracy of the models was improved by more spectral variables being involved in the models (**Figure 4**), with the highest *R^2^* value of 0.887 given by the model based on the four growth stages. However, the best models based on variables at three growth stages (*R^2^* = 0.887), and even at two growth stages (*R^2^* = 0.869), were almost equivalent to the model based on the four growth stages, especially when those models involved variables at the ripening stage. Furthermore, *R^2^* values of models that involved variables at the ripening stage were always higher than those of models without variables at the ripening stage, further indicating the key role of the ripening stage for AC estimation.

Models that had variables at the ripening stage were further tested using a validation dataset, except the model developed by the four growth stages, which was just slightly better than those using variables less than four. The following three models with high stability and accuracy in both calibration and validation were identified, and their expressions were as in Eqs. 2–4:


(2) $$AC_{S}=217.83-207.03*RVI_{\left[580,568](Ripening\right)}$$

(3) $$AC_{S}=58.6+84.45*RVI_{\left[584,592](Heading\right)}-133.75*RVI_{\left[580,568](Ripening\right)}$$

(4) $$AC_{S}=106.17+40.99*RVI_{\left[584,592](Heading\right)}-850.84*DVI_{\left[664,672](Filling\right)}-135.401*RVI_{\left[580,568](Ripening\right)}$$

where *AC_s_* estimated AC by models using spectral variables alone.

As expected, the best single growth stage-based model was constructed by RVI at the ripening stage (Eq. 2). Three involved vegetation indices were all constructed by reflectance in the green and red regions.

### Estimation of amylose content based on textural information alone

#### Relationship between amylose content and textural measures

As the heading, filling, and ripening stages are significant to the estimation of AC, the relationship between AC and textural measures, including eight textural statistics (TS), textural index in differential type [difference textural index (DTI)], and ratio type [ratio textural index (RTI)], at these three stages was analyzed (**Table 6**).

**Table 6:** Correlation coefficients (*r*) for the relationship between amylose content and textural measures.

Growth stage	TS		DTI		RTI	
	Variable	*r*	Variable	*r*	Variable	*r*
Heading	CON_704_	-0.481**	DTI_VAR[840,864]_	-0.768**	RTI_CON[840,848]_	-0.723**
Filling	COR_704_	0.700**	DTI_DIS[504,720]_	0.735**	RTI_HOM[504,720]_	-0.730**
Ripening	CON_650_	-0.708**	DTI_CON[688,672]_	-0.828**	RTI_HOM[680,656]_	-0.824**

** denotes significance at the 0.001 probability level (when the numbers of samples n = 80, P_0.001_ = 0.356). TS, textural statistics; DTI, difference textural index; RTI, ratio textural index.

The best correlations between AC and various textural measures were all significant at the 0.001 probability level, and all happened at the ripening stage, which were similar to the correlations between AC and spectral variables. However, contrary to the significant improvement in the correlation between AC and spectral indices caused by transforming spectral reflectance into vegetation indices, increasing the significance level in textural indices was less, with the correlation between either DTI or RTI and AC slightly better than those between TS and AC, indicating the great potential of the selected TS to be used directly in AC estimation.

The maximum correlation coefficient (*r*_max_) was obtained by RTI at the ripening stage (*r* = 0.828), and *r*_max_ for each type of textural measures occurred at the ripening stage, again proving the significant role of the ripening stage for the estimation of AC (**Table 6**). Meanwhile, 704 nm was involved in CON_704_ at the heading stage and COR_704_ at the filling stage, and 650 nm was in CON_650_ at the ripening stage, indicating the importance of textural measures at 704 and 650 nm for the estimation of AC based on textural measures of UAV spectral images. In fact, the intermediate results showed that CON_704_, DIS_704_, HOM_704_, and VAR_704_ at the filling stage and CON_650_, VAR_650_, DIS_650_, HOM_650_, and ENT_650_ at the ripening stage also had significant correlation with AC.

Four textural measures that significantly correlated to AC were HOM, DIS, CON, and VAR, and textural indices with the best performance were mainly constructed by those four types of textural measures. Actually, they characterize similar features of images from different aspects. The significant relation between textural measures and AC can partly be explained by ambient temperature, which is a key impact factor for plant growth. Previous studies have proven that rice ACs are affected by environmental factors, particularly ambient air temperature (Larkin and Park, 1999; Bergman, 2019), which also influences plant traits and phenotypic parameters (Feng et al., 2021), including yield, plant height, LAI, and chlorophyll. Differences in these parameters and in AC will in turn lead to changes of textural features in UAV hyperspectral images and thus influence their relation. The role of textural information in improving rice yield estimation has been demonstrated by Wang et al. (2021). AC is an important component of yield; its great correlation with textural measures is partly attributed to the good correlation between yield and textural measures. Additionally, the correlation of textural measures with spectral variables also contributed to the significant relation between textural measures and AC. Despite these possible reasons, understanding the relation of textural information in UAV hyperspectral images with rice AC from cause-and-effect aspects is insufficient and further exploration is needed.

#### Estimation models based on textural variables alone

The models using textural measures at one (Eq. 5), two (Eq. 6), and three growth stages (Eq. 7) were constructed, and the formulas of the models (Eqs. 5–7) with the highest coefficient of determination were as follows:


(5) $$AC_{T}=58.689-43.593*RTI_{HOM[680,656](Ripening)}$$

(6) $$AC_{T}=48.836-33.429*RTI_{HOM[656,680](Ripening)}-31.998*DTI_{VAR[528,608](Filling)}$$

(7) $$AC_{T}=46.876-30.409*RTI_{HOM[656,680](Ripening)}-29.157*DTI_{VAR[528,608](Filling)}-2.388*CON_{704(Heading)}$$

where *AC_T_* is the estimated AC derived from models constructed by textural measures alone.

Similar to the model using spectral variables alone, the single growth stage-based model was constructed by textural indices at the ripening stage. The model (Eq. 7) based on textural measures at three growth stages gives the highest *R^2^* of 0.843.

### Estimation of amylose content based on combined spectral and textural information

Models based on combined spectral and textural variables were established on the basis of the aforementioned results. As noticed, the accuracies of the models were generally improved when more independent variables were involved. To make the comparison of different models more persuasive, the number of independent variables in models based on combined spectral and textural variables was also limited to 3, consistent with the independent variables in models using spectral or textural measures alone.

The spectral and textural variables in the simplest model (Eq. 8) both came from the ripening stage, further demonstrating the role of variables at the ripening stage for AC estimation. The *R^2^* values of models using three variables (Eqs. 9–11) were very close to each other, with the maximum of 0.913 and the minimum of 0.896 almost indistinguishable (**Table 7**). The model based on spectral indices RVI at the ripening stage and two textural indices (Eq. 9), i.e., RTI at the ripening stage and DTI at the filling stage, was slightly better than the two other models, indicating the usefulness of textural information. Additionally, two variables in Eq. 8 were both RVI-type indices, highlighting that the method of translating spectral reflectance or TS into ratio type was a good choice in forming indices.

**Table 7:** Formula and coefficient of determination (*R^2^*) of the models based on combined spectral and textural variables.

Numbers of growth stage	Model expressions		*R^2^*
**One**	$AC_{S}\&T=134.485-143.466*RVI_{\left[580,568\right](Ripening)}+21.203*RTI_{HOM\left[680,656\right](Ripening)}$	**Eq. 8**	**0.868**
**Two**	$AC_{S\&T}=109.788-114.44*RVI_{\left[580,568\right](Ripening)}+18.631*RTI_{HOM\left[680,656\right](Ripening)}+3.456*DTI_{DIS\left[504,720\right](Filling)}$	**Eq. 9**	**0.913**
	$AC_{S\&T}=113.626-116.7*RVI_{\left[580,568\right](Ripening)}+16.859*RTI_{HOM\left[680,656\right](Ripening)}+938.708*DVI_{\left[664,672\right](Filling)}$	Eq. 10	0.912
	$AC_{S\&T}=36.816-103.679*RVI_{\left[580,568\right](Ripening)}+16.858*RTI_{HOM\left[680,656\right](Ripening)}+60.857*RVI_{\left[584,592\right](Heading)}$	Eq. 11	0.896

AC_S&T_ are estimated amylose content by models constructed by combined spectral and textural measures. TS, textural statistics; DTI, difference textural index; RTI, ratio textural index. The equations in bold are the adopted models.

### Validation and comparison of amylose estimation models

To verify the robustness of estimation models and identify the model with high accuracy and simple construction, all established models, including those based on spectral variables alone, textural variables alone, and combined spectral and textural variables, were all tested by calibration and validation datasets (**Table 8**).

**Table 8:** Model test results by the calibration and validation datasets.

Criteria	Numbers of Growth stage	Calibration dataset			Validation dataset		
		Spectral	Textural	Spectral and textural	Spectral	Textural	Spectral and textural
***R^2^***	One	0.795 (Eq. 2)	0.684 (Eq. 5)	**0.868 (Eq. 8)**	0.702	0.678	**0.791**
	Two	0.854 (Eq. 3)	0.805 (Eq. 6)	**0.913 (Eq. 9)**	0.767	0.797	**0.826**
	Three	0.877 (Eq. 4)	0.843 (Eq. 7)	/	0.775	0.824	/
**RMSE (%)**	One	1.3	1.61	**1.04**	1.72	1.64	**1.37**
	Two	1.1	1.27	**0.844**	1.42	1.28	**1.22**
	Three	1.01	1.14	/	1.44	1.18	/
**MAPE (%)**	One	8.04	9.64	**6.42**	9.63	10.43	**7.76**
	Two	6.72	7.30	**4.70**	7.51	7.68	**7.08**
	Three	5.73	6.59	/	8.01	7.11	/

The optimum values of criteria are in bold.

First, the numbers of growth stage that were involved in the models were compared, as the fewer growth stages in the models, the easier the data to be obtained, and thus the more likely the model to be used extensively. The priority was given to the model with simpler construction when accuracy was similar.

The model (Eq. 8) based on combined spectral and textural variables at one growth stage, i.e., the ripening stage, with *R^2^* of 0.868 and 0.791, RMSE of 1.04 and 1.37, MAPE of 6.42% and 7.76% for the calibration and validation datasets, respectively, was obviously superior to the models based on spectral variable (Eq. 2) or textural variable alone (Eq. 5) at one growth stage, even better than those constructed by variables at two growth stages (Eqs. 3, 6) and by variables at three growth stages (Eq. 4). Among all tested models, the model (Eq. 9) constructed by combined spectral and textural variables with three variables at two growth stages gives the best performance in all three test criteria, better than models using variables at three growth stages. The superiority of combining spectral and textural measures to spectral or textural variables alone in accuracy for the estimation of crop parameters has been demonstrated by some previous research (Yue et al., 2019; Zheng et al., 2020; Wang et al., 2021), which generally attributed the outperformance partly to the complementary information between textural and spectral variables.

**Figure 5:**
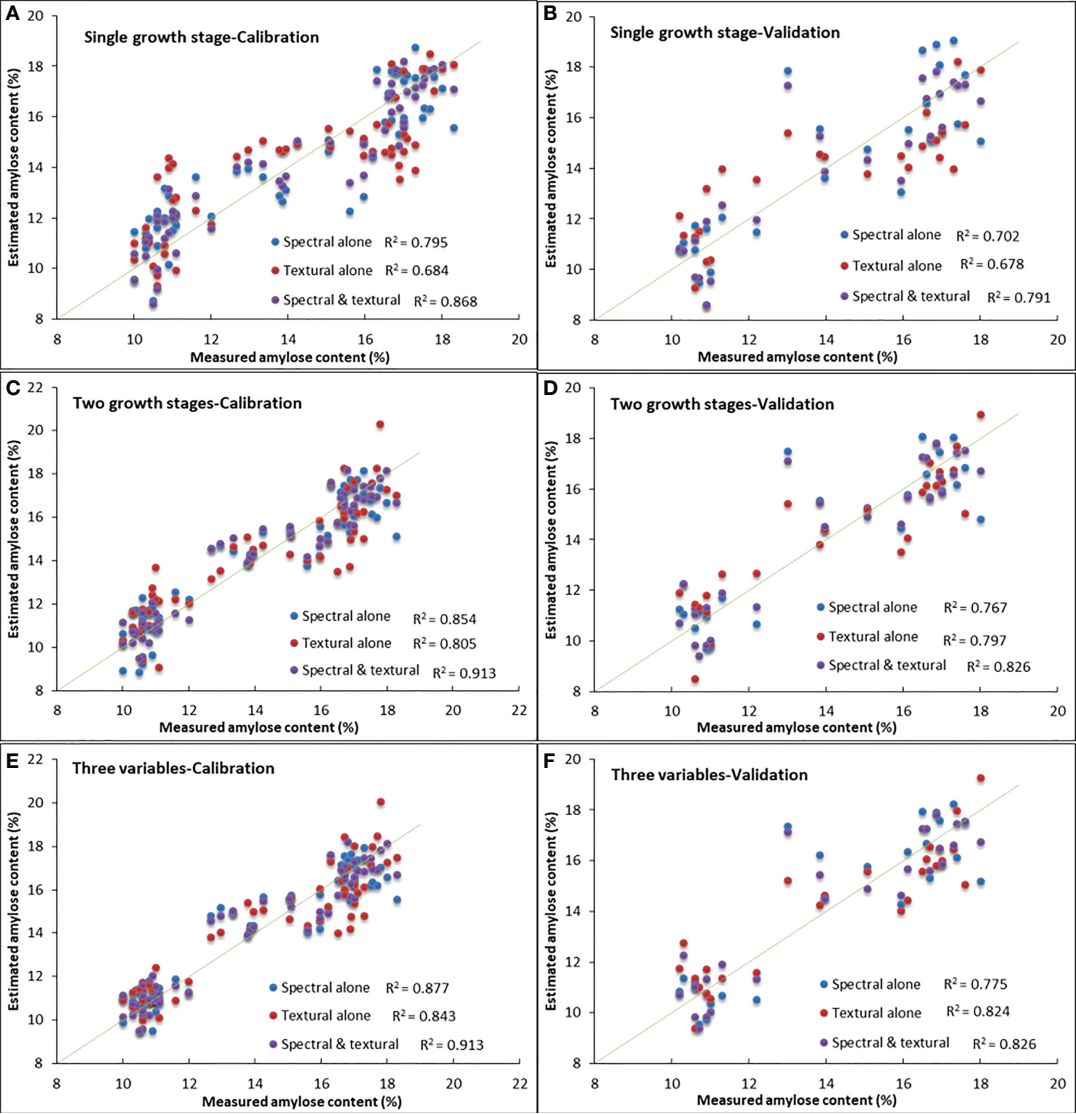
Measured vs. estimated amylose content derived from models based on spectral variables alone, textural variables alone, and combined spectral and textural variables. Panels **(A**, **C**, **E)** are results from the calibration dataset. Panels **(B**, **D**, **F)** are results from the validation dataset.

The intercomparison was carried out among models based on spectral variables alone, textural measures alone, and combined spectral and textural variables. As models based on spectral variables alone (Eq. 4) or textural variables alone (Eq. 7) had three variables at three growth stages and the model (Eq. 9) based on spectral and textural variables had three variables at two growth stages, **Figures 5E, F** were noted as three variables instead of three growth stages.

The overall performance of models based on spectral and textural variables was always superior to those based on spectral or textural variables alone, and results for the calibration dataset were better than those for the validation dataset (**Figure 5**). For models (Eqs. 2, 5, and 8) based on variables at one growth stage (**Figures 5A, B** **)**, i.e., the ripening stage, textural-based model (Eq. 5) showed the problem of overestimation when AC was lower than 15% but underestimation when AC was higher than 15%, and the spectral-based model tended to overestimate when AC was below 13%. The same tendency can be found in **Figures 5C, D**, in which two growth stages were involved. However, the spectral and textural-based model (Eq. 9) was likely to have the ability to adjust the overestimation or underestimation of AC in spectral-based or textural-based models, thus making it better.

Furthermore, the fitness between measured and estimated AC visually demonstrated the aforementioned conclusion that spectral and textural-based model (Eq. 8) using variables at the ripening stage alone can give a similar accuracy to spectral-based or textural-based models that have one or two growth stages involved, indicating the superiority of the method using combined spectral and textural variables for AC estimation in simplifying model structure and data acquisition. Additionally, no matter whether the comparison was carried out among the same number of growth stages (**Figures 5C, D** **)** or among the same number of independent variables (**Figures 5E, F** **)**, the spectral and textural-based model (Eq. 9) always gave the best estimation of AC, proving the great potential of combined spectral and textural information in UAV hyperspectral images for rice AC estimation. The test results using the validation dataset were generally very similar to those of the calibration dataset, indicating good stability of the established models.

#### Discussion

The present study makes the first attempt of using UAV technique for rice AC monitoring, and results are encouraging. However, as seen, the above established models were based on correlations between spectral or textural information and laboratory-determined AC; they are empirical, not based on cause and effect. Previous research showed that crop AC is affected by environmental factors, particularly ambient air temperature (Ziska et al., 1997; Madan et al., 2012); even the same cultivar grown in different environments may vary by up to 6% in AC (Juliano and Pascual, 1980). In this study, five observed rice cultivars grew in small experimental plots, where the growing conditions have no evident differences except for N fertilization, making the variation in AC for a certain rice cultivar small; this means that the efficiency of the results obtained herein still needs more verification at a larger scale.

Additionally, textural features of images are significantly affected by spatial resolution (Liu et al., 2018a; Liu et al., 2018b), which was partly determined by the flight altitudes of UAVs. The different flight altitudes will make the spatial resolution different and thus lead to the changes of textural features. Here, the flight altitude for image acquisition was fixed at 200 m, with a ground spatial resolution of 0.13 m, so the effects of differences in spatial resolution caused by flight altitudes on values of textural measures were not considered.

Furthermore, the method for GLCM construction and the window size for the calculation of textural measures may also cause differences in the final results (Sarker and Nichol, 2011; Zheng et al., 2019). Although the methods for GLCM development and TS calculation here were adopted by trials and errors, whether the plan is suitable for other cases is unclear. Consequently, the reliability and robustness of these empirical relations and models proposed here need to be evaluated and verified by more rice cultivars growing under varied environmental conditions. In addition, more methods for model development need to be tested to make the application of UAV technique in rice AC monitoring more confident.

## Conclusions

In this study, the potential of spectral and textural information in UAV hyperspectral images for rice AC was tested. The following conclusions can be drawn:

1) Spectral and textural variables at the ripening stage are crucial to rice AC estimation, and the most sensitive bands to rice AC are mainly located in the green and red regions.

2) Performances of models based on spectral variables or textural variables alone are equivalent, with the best estimation given by models constructed by variables at the ripening, filling, and heading stages.

3) In comparison to models developed by spectral or textural variables alone, models based on combined spectral and textural variables can give better estimation of AC with less growth stages involved.

It can be inferred that using spectral and textural information in UAV-based hyperspectral images has great potential to obtain the knowledge of AC of a standing crop in advance and thus would not only provide scientists and technicians alternative or complementary information to define the targeted rice market class in a rapid and non-destructive way but also provide opportunities to manage grain harvest differently.

## Data availability statement

The raw data supporting the conclusions of this article will be made available by the authors, without undue reservation.

## Author contributions

FW: Conceptualization, Methodology, Investigation, Resources, Supervision, Writing- Original draft preparation, Projection administration, Funding acquisition; QY: Formal analysis, Data curation, Writing- Original draft preparation, Writing - Review and Editing, Visualization, Funding acquisition; LX: Software, Formal analysis, Data curation, Investigation; XY, TX, JZ, JL and SC: Validation, Investigation. All authors contributed to the article and approved the submitted version.

## Funding

This study is supported by the Third Xinjiang Scientific Expedition Program (2021xjkk1400) and National Natural Science Foundation of China (41871328).

## Acknowledgments

The assistance given by colleagues from the Institute of Applied Remote Sensing & Information Technology, Zhejiang University, and local farmers is highly appreciated.

## Conflict of interest

The authors declare that the research was conducted in the absence of any commercial or financial relationships that could be construed as a potential conflict of interest.

## Publisher’s note

All claims expressed in this article are solely those of the authors and do not necessarily represent those of their affiliated organizations, or those of the publisher, the editors and the reviewers. Any product that may be evaluated in this article, or claim that may be made by its manufacturer, is not guaranteed or endorsed by the publisher.
